# Establishing a Berry Sensory Evaluation Model Based on Machine Learning

**DOI:** 10.3390/foods12183502

**Published:** 2023-09-20

**Authors:** Minghao Liu, Minhua Liu, Lin Bai, Wei Shang, Runhan Ren, Zhiyao Zhao, Ying Sun

**Affiliations:** 1 School of Artifical Intelligence, Beijing Technology and Business University, Beijing 100048, China; 2230602061@st.btbu.edu.cn (M.L.); liuminhua@btbu.edu.cn (M.L.); zhaozy@btbu.edu.cn (Z.Z.); 2 College of Chemistry and Materials Engineering, Beijing Technology and Business University, Beijing 100048, China; 2230402043@st.btbu.edu.cn (L.B.); 2130041010@st.btbu.edu.cn (W.S.); 2230401016@st.btbu.edu.cn (R.R.); 3 School of Light Industry, Beijing Technology and Business University, Beijing 100048, China

**Keywords:** sensory evaluation, blueberry, preservation, food shelf life, particle swarm arithmetic, support vector machines

## Abstract

In recent years, people’s quality of life has increased, and the requirements for fruits have also become higher; blueberries are particularly popular because of their rich nutrients. In the blueberry industry chain, sensory evaluation is an important link in determining the quality of blueberries. Therefore, to make a more objective scientific evaluation of blueberry quality and reduce the influence of human factors, on the basis of traditional sensory evaluation methods, machine learning is introduced to establish a support vector regression prediction model optimized by the particle swarm algorithm. Ten physical and chemical flavor indices of blueberries (such as catalase, flavonoids, and soluble solids) were used as input data, and sensory evaluation scores were used as output data. Three different predictive models were applied and compared: a particle swarm optimization support vector machine, a convolutional neural network, and a long short-term memory network model. To ensure reliability, the experiments with each of the three models were repeated 20 times, and the mean of each index was calculated. The experimental results showed that the root mean square error and mean absolute error of the particle swarm optimization support vector machine were 0.45 and 0.40, respectively; these values were lower than those of the convolutional neural network (0.96 and 0.78, respectively) and the long short-term memory network (1.22 and 0.97, respectively). Hence, these results highlighted the superiority of the proposed model when sample data are limited.

## 1. Introduction

Native to North America, blueberry is a collective name for the green fruit group of plants in the Rhododendron family producing small berries [1]. Blueberries are increasingly found worldwide. Their flesh is rich in anthocyanins, low in sugar, low in fat, and high in antioxidants. The International Food and Agriculture Organization lists blueberries as one of the five major healthy foods for humans. It is also known as the “king of berries” and is favored by people [2]. The blueberry fruit tree species has high economic value and broad development prospects [3]. In recent years, China’s blueberry planting area and output have generally shown a year-over-year growth trend. In 2020, China’s blueberry planting area was 66,400 hectares, showing a year-over-year increase of 10%; moreover, production was 347,200 tons, which was up 64% year-over-year [4]. However, blueberry fruits are soft and susceptible to pathogenic bacteria, resulting in quality deterioration and affecting their storage, circulation, and sale [5]. Therefore, the requirements related to the transportation and storage conditions of blueberries have increased; therefore, an effective method is needed to predict the quality changes of blueberries and thus prevent them from spoiling during storage.

Currently, there are not many sensory evaluation methods for fruits and vegetables. Some are based on manual evaluation, such as the study of the color and leaf shape of lettuce varieties and taste [6]. Sensory evaluation of apples is commonly made by evaluating apple crispness and hardness [7]. However, these manual evaluation methods require knowledgeable and experienced assessment experts and are easily affected by subjective factors; therefore, evaluation results can deviate from the actual situation. Other sensory evaluation methods for fruits and vegetables utilize sensors and technology, such as electronic nose technology and spectroscopy. For example, electronic nose technology is used to assess the freshness of fresh-cut green bell peppers [8], while freshness can be assessed via spectral analysis [9]. Compared with manual evaluation methods, these methods are more objective and reliable. However, the technologies employed are not perfect. For instance, the selectivity and limitations of sensors prevent their use for all objects. Moreover, to prevent sensors from being affected by environmental factors such as temperature and humidity, there are high requirements for the detection environment.

There are relatively few sensory evaluation methods for blueberries. Most of the research on blueberries focuses on one or more of their ingredients or processed products. This includes optimizing the extraction process of blueberry anthocyanins and studying their effects on immunomodulatory activity [10], extracting the polyphenol components in blueberries and studying their applications in biological experiments [11], observing the fermentation process during the production of blueberry wine, and studying the wine’s final color and anthocyanin content [12]. Research has also focused on changing the freezing conditions of blueberry juice to observe the effect of freezing on the nutritional content of the juice [13].

In addition, only a small percentage of blueberry studies are on sensory evaluation methods. Many researchers have studied the effects of storage methods on blueberry quality. For example, the effects of different packaging on the postharvest storage quality and antioxidant activity of blueberry fruits under low-temperature conditions were studied, and suitable storage and packaging methods were explored to improve blueberry quality [14]. It was found that blueberry leaf extract has good bacteriostatic activity and thus can be used to coat fresh blueberries to extend their shelf life and maintain their nutrients [15]. Other studies have focused on several indicators of blueberries using principal component analysis and cluster analysis and then conducted sensory evaluation of fresh blueberries. For example, a physical property analyzer was used to identify the blueberry fruit: 10 texture parameters were obtained, and then blueberries were evaluated and classified using principal component analysis and cluster analysis [16]. However, these sensory evaluation methods still have some drawbacks. They rely too much on data, have high requirements for evaluators, and lack more comprehensive sensory evaluation methods. Therefore, it is of great significance to study the changes in different physical and chemical indices in blueberries, link physical and chemical index parameters with the sensory evaluations of experts, and establish a comprehensive measurement and prediction model of blueberry sensory evaluation on the basis of traditional sensory evaluation methods.

A support vector machine (SVM) is a machine learning algorithm based on statistical learning theory and has a strong theoretical foundation. Moreover, the extreme solution that the SVM can guarantee is the global optimal solution rather than the local minimum value. This leads to the excellent small-sample learning ability and good generalizability of the SVM; thus, it can be used in classification, prediction, regression estimation, and other fields [17,18]. For example, relative characteristics and multivariate SVM are used to predict the remaining life of rolling bearings [19]. SVMs are also used to improve remote sensing image classification [20]. SVMs can not only characterize the nonlinear relationship between multiple characteristics of sample input data and target output data, but also have high accuracy and good stability [21]. The main factors affecting the accuracy, stability, and generalization of SVMs are the penalty factor c and relaxation factor g [22]. The selection of these core parameters will directly affect the prediction accuracy and classification performance of an SVM; therefore, the optimization of these parameters is critical. Considering this, we use optimization algorithms to optimize parameters, and in this paper, we use particle swarm optimization algorithms (PSO).

## 2. Materials and Methods

### 2.1. Materials and Reagents

The blueberry fruit variety in this study was a Gaoshan blueberry picked in April 2023 from Qingdao, Shandong, China. Superoxide dismutase (SOD), catalase (CAT), ascorbate peroxidase (APX), peroxidase (POD), polyphenol oxidase (PPO), and a hydrogen peroxide (H_2_O_2_) determination kit were supplied by Nanjing Jiancheng Bioengineering Research Institute Co., Ltd. in Nanjing, Jingsu Province, China. Normal saline was provided by Shijiazhuang Four Medicine Co., Ltd. in Shijiazhuang, Hebei Province, China.

### 2.2. Instruments and Equipment

We used a Bio-tek microplate reader (SYNERGY Inc., New York, NY, USA), spectrophotometer (Agilent Technologies Inc., Beijing, China), 3K15 high-speed refrigerated centrifuge (SIGMA, Osterode, Lower Saxony, Germany), T-403 digital electronic balance (Beijing Sartorius Instrument System Co., Ltd., Beijing, China), DF-101S constant temperature collector heating magnetic stirrer (Gongyi Yuhua Instrument Co., Ltd., Zhengzhou, Henan Province, China), and an F-80C ice machine (Beijing Bowei Xingye Technology Development Co., Ltd., Beijing, China) for this study.

### 2.3. Methods

#### 2.3.1. Blueberry Sample Design

Fruits with the same volume, color, and ripeness were chosen. The blueberries were freshly picked Gaoshan blueberries from Qingdao, Shandong (eight ripe, dark color, single fruits, weight 15 ± 1 g), without disease, pests, or mechanical injuries, and were tested at room temperature.

#### 2.3.2. Determination of Physical and Chemical Indices

Assays were performed according to the methods provided in the SOD, CAT, APX, POD, PPO, and H_2_O_2_ assay kits. According to m (blueberry weight):m (normal saline weight) = 1:9, a 10% tissue homogeneous slurry under ice water bath conditions was prepared, and the supernatant was obtained after 3500 rpm/separation of the core for 10 min. The kit steps were followed to prepare the experimental tube and control tube, and then reagent 1 was added to reagent 4. After homogenization, the supernatant was obtained after 10 min of 3500 rpm/separation. At the corresponding wavelength, the assay was performed using a microplate reader [23]. The entire process was repeated three times in parallel to calculate the enzyme activity.

The method for determining flavonoids is as follows [24]: first, 10 mg of rutin was weighed, 10 mL of absolute ethanol was added, and a 0.1 mg/mL standard solution was prepared. Next, 5 g of blueberry samples was weighed and placed in a 50 mL brown volumetric flask, and 35 mL of absolute ethanol was added. The sample was sonicated for 60 min, the volume was scaled with absolute ethanol, and the mixture was shaken well. Then, 25 mL of the treated solution was transferred to a 50 mL centrifuge tube and centrifuged at 6000 rpm for 10 min, and the supernatant was set aside for later use. Samples of 0.00 mL, 1.00 mL, 2.00 mL, 3.00 mL, 4.00 mL, and 5.00 mL of rutin standard solution were aspirated and placed in a 25 mL cuvette tube, and water was added to reach a total solution volume of 10 mL. Then, 1.0 mL of sodium nitrite (50 g/L) was added to the samples. The solution was left to rest for 6 min, then 1.0 mL of aluminum nitrate solution (100 g/L) was added and left to rest for another 6 min. Finally, 4.0 mL of sodium hydroxide solution (40 g/L) was added, water was added to the scale, and the resulting solution set aside for 15 min. Using a 1 cm cuvette, the zero point was adjusted with a reagent blank, and the absorbance at a wavelength of 510 nm was determined. Using the absorbance as the ordinate and rutin mass as the abscissa, the standard curve was drawn. Next, 2.0 mL of the test solution was pipetted and transferred into a 25 mL colorimetric tube, and water was added to reach a total volume of 10 mL. The absorbance at a wavelength of 510 nm was determined.

The method to determine total polyphenols is as follows [25]: first, 10 mg of gallic acid was placed in a 100 mL brown volumetric flask, and water was added to form a 0.1 mg/mL standard solution. A total of 10 g of the blueberry sample was weighed and placed in a 100 mL brown volumetric flask, then an appropriate amount of water was added. The mixture was sonicate for 60 min and cooled to room temperature. Next, the volume was set with water to the scale, shaken well, and 30–35 mL of the treated solution was added to a 50 mL centrifuge tube. The solution was centrifuged at 6000 rpm for 15 min, and the supernatant was set aside for later use. Samples of 0.20 mL, 0.40 mL, 0.60 mL, and 0.80 mL of the standard solution were pipetted into a 10 mL volumetric flask, and 3–4 mL of water was added to each sample. Next, 0.5 mL of forinphenol test solution was added to each flask. Within 1–8 min, 1.5 mL of Na_2_CO_3_ solution (20.0 g/100 mL of aqueous solution) was added to each flask. Gallic acid concentrations of 0.002 mg/mL, 0.004 mg/mL, 0.006 mg/mL, and 0.008 mg/mL were obtained by volumetrically scaling the solution with water, and each volumetric flask was placed in a 30 °C water bath for 2 h. At the same time, a blank solution was prepared, the absorbance was measured at 760 nm (within 10 min), and the regression curve was drawn with the absorbance as the ordinate and the concentration as the abscissa. Finally, 0.2 mL of the test solution was accurately pipetted and added to a 10 mL volumetric flask. Then, 3–4 mL of water was added to the test solution and zeroed with a blank solution. Finally, the absorbance at 760 nm was determined (within 10 min).

To determine the pH value, the edible part of the blueberry was pulped. Equal amounts of pulp and Watsons water were mixed well. The pH was measured five times using a Mettle Toledo FE28-standard pH meter, and the average was taken.

The determination of soluble solid content (SSC) was as follows [26]: the blueberries were partially beaten, and the juice was extracted using four layers of gauze. The SSC was measured three times using an Abbemat 500 automatic refractometer.

#### 2.3.3. Sensory Evaluation

The sensory evaluation team consisted of five women and five men (aged 25–55 years). They were able to distinguish between basic tastes (bitter, sweet, sour, salty, and umami) and had experience in sensory rating and flavor analysis of fruits and vegetables. Twenty expert group members (10 males and 10 females, aged from 25 to 55) with sensory experiment and quantitative description analysis experience were recruited from our laboratory. They were trained for 4 weeks (20 min/day) to describe the taste characteristics of blueberry samples, including the appearance, hardness, color, aroma, and taste as evaluation indexes, and distinguish their differences. Finally, 10 experts (5 men and 5 women, ages 25 to 55) who correctly distinguished the tastes were selected. 

The descriptive terms and their evaluation criteria were defined as follows: (1) acidity: 0.05 g citric acid/100 mL water = acidic 10, 0.1 g citric acid/100 mL water = acidic 20; (2) sweetness: 2 g sucrose/100 mL water = sweetness 10, 4 g sucrose/100 mL water = sweetness 20; (3) bitterness: 0.00075 g quinine/100 mL of water = bitter 10, 0.0015 g quinine/100 mL of water = bitter 20; (4) appearance: blueberry fruit is complete, no mechanical damage = appearance 20; (5) hardness: high hardness, no rot = hardness 20; (6) color: deep, uniform color = color 20; (7) aroma: blueberry has a rich aroma, no peculiar smell, no astringency = aroma 20; and (8) taste: a combination of different flavors. Based on these sensory assessment criteria, the panel members received six training sessions for 2 weeks. Finally, all the group members were able to identify these descriptors and use them consistently.

The cleaned blueberry fruit samples were placed in a randomly numbered white plate and presented to 10 sensory evaluators (5 males and 5 females, 25 to 55 years of age) for evaluation based on the appearance and hardness. The appearance, hardness, color, aroma, and taste were used as the evaluation indicators. Each indicator had a full score of 20 points, for a total of 100 points. Table 1 shows the scoring rules for sensory evaluation. Sensory evaluation was performed in a sensory panel room at 22 ± 2 °C with 40–80% humidity. All the panelists rinsed their mouths with boiled water, and each sample was tasted after a rest period of 15 s. The results evaluated by each single panelist differed by <20%. To avoid fatigue and carryover effects, the panel members were asked to rinse their mouths with 50–60 mL of drinking water between tests of two different samples. No eating, drinking, or smoking was allowed 1 h before the sensory assessment. We collected all the score cards at the end of each evaluation and calculated the average of all the descriptors given by the panel members across three replicate experiments. We also conducted multivariate statistical analyses. 

#### 2.3.4. Data Processing

The physical and chemical indices were determined, and sensory evaluations were conducted every 0.5 days and measured for 6 days. Three replicate experiments were conducted in parallel each time. A total of 36 experimental samples were measured. The physical and chemical indices and sensory evaluation scores of some of the samples are shown in Table 2.

## 3. Model Construction

**Figure 1:**
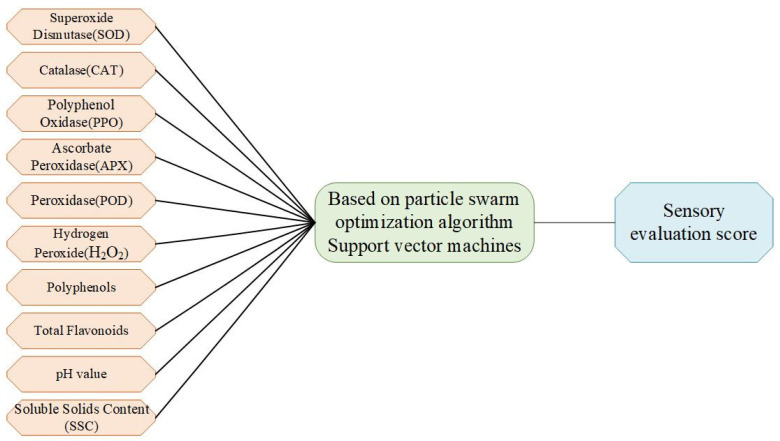
Blueberry sensory evaluation model structure.

In this paper, an SVM optimized using PSO is used for the sensory evaluation prediction of blueberries. The measured blueberry data are normalized, and the three sets of parallel data that were experimentally obtained are used as the training set. The mean of the three sets of parallel data is used as the test set to verify the estimation results of the model. We used 10 physical and chemical flavor indices as the input data and blueberry sensory evaluation scores as the output data. A structure diagram of the blueberry sensory evaluation prediction model is shown in Figure 1.

### 3.1. SVM Model

**Figure 2:**
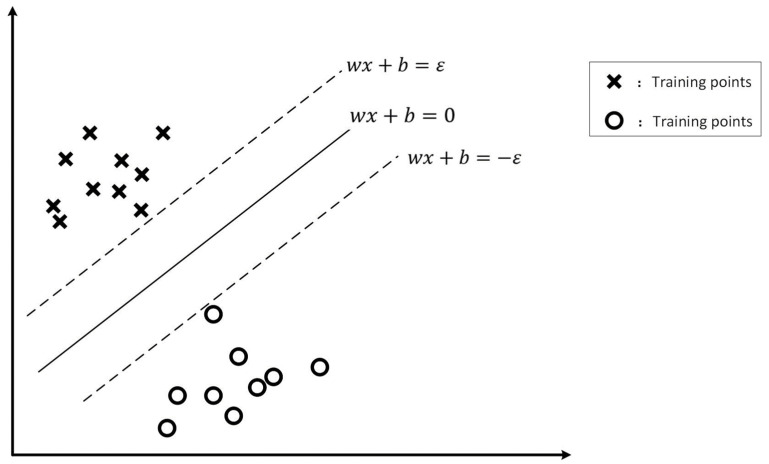
Schematic of the principle of two-dimensional spatial linear classification.

SVMs are novel statistical learning methods for small samples [27]. They are used for classification and regression analyses. SVMs have two important ideas: structural risk minimization and the application of kernel functions. Figure 2 shows a linear classification problem in a two-dimensional space; here, the purpose of the classification is to find a straight line to distinguish between two types of samples. SVM classification should not only separate the samples without error but also maximize the class interval to ensure generalizability [28]. In this paper, we also find an optimal hyperplane so that all the data samples are as close to the hyperplane as possible. Hyperplanes can be represented as follows:

(1) $$f(x)=\omega^{T}x+b$$

where $\omega^{T}$ is the weight vector transpose and b is the bias.

The optimal regression hyperplane problem can be transformed into a quadratic programming problem and finally into a convex optimization problem, which can ensure the global optimality of the algorithm and prevent it from falling into local minimum points [29]. The optimization problem is as follows:

(2) $$min\frac{1}{2}||\omega |{|}^{2}+c\sum_{i=1}^{l}\left(\xi_{i}+\xi_{i}^{*}\right)$$

(3) $$s.t.\left\{\begin{array}{c} y_{i}-\omega^{T}\varphi (x_{i})-b\leq \varepsilon +\xi_{i}\\ \omega^{T}\varphi (x_{i})+b-y_{i}\leq \varepsilon +\xi_{i}^{*},i=1,2\cdots l\\ \xi_{i},\xi_{i}^{*}\geq 0 \end{array}\right.$$

where c is the penalty factor, which indicates the degree of punishment for the sample when the specified range ε allowed for error is exceeded. Its value reflects the judgment of the importance of the two parts in Formula (2). $\xi_{i}$ and $\xi_{i}^{*}$ are relaxation variables that reflect the fitting error and reduce the requirements for hyperplanes, $\varphi (x_{i})$ is a nonlinear transformation that maps data to a high-dimensional space, and ε is an insensitive parameter. The width of the insensitive band is determined, and loss is not calculated for samples falling into it; that is, only the samples that fall outside the insensitive band will affect the SVM. Finally, the minimized total loss and maximized interval are used to determine the optimized model [30].

The SVM has four kernel functions: the radial basis function, linear function, polynomial function, and activation function. Radial basis functions have a strong nonlinear modeling ability, strong flexibility generalization abilities, few hyperparameters, and are widely used; therefore, radial basis functions are used in this paper [31]. The above quadratic programming problem is solved using the Lagrange multiplier method, and the decision function is given as follows:

(4) $$f(x)=\sum_{i=1}^{l}(a_{i}-{a_{i}}^{*})K(x,x_{i})+b$$

where $a_{i}$ and $a_{j}$ are Lagrange multipliers.

$K(x_{i},x_{j})=\varphi {\left(x_{i}\right)}^{T}\varphi (x_{j})$ is used to describe the inner product of a high-dimensional feature space.

The parameter values also have a significant influence on the SVM model. The selection of appropriate parameters can provide the SVM model with good learnability and generalizability [32]. The main parameters of the SVM model are the penalty factor c and the parameter g of the radial basis kernel function. The penalty factor c plays a role in the complexity and stability of the model and controls the trade-off between sample bias and generalizability. When c is very small, the penalty for samples outside the ε channel is small, which will lead to an increase in the training error and a greater structural risk. When c becomes larger, the training error decreases and the fit of the data increases, but if c is too large, it will lead to overfitting. Meanwhile, the kernel function parameter g is used to define the high-dimensional feature structure, which indicates the degree of correlation of each support vector in the high-dimensional space. When g is small, there is a loose connection between support vectors, resulting in higher complexity and poor generalizability of the training model. When g increases, the influence between support vectors increases, but when g is too large, the model accuracy is difficult to guarantee [33]. Therefore, to obtain the SVM model with the highest accuracy, the PSO algorithm is used to optimize parameters c and g.

### 3.2. Particle Swarm Optimization

PSO is an evolutionary computing technique that was first proposed by Eberhart, a computational intelligence researcher, and Kennedy, a psychologist [34]. The PSO algorithm originates from the study of bird group activities and abstracts each bird into a particle without mass and volume by simulating feeding behavior. It regards the process of finding the optimal solution to a problem as the process of birds looking for food. PSO allows us to solve complex optimization problems. PSO algorithms have the advantages of a simple concept, low computational requirements, fast solutions, and strong global search abilities; therefore, they are widely used for function optimization, pattern recognition, and in other fields [35].

In PSO, each member of the population is called a particle and represents a potentially feasible solution; meanwhile, the location of food is considered the global optimal solution. The swarm searches for the global optimal solution in the D-dimensional solution space, and each particle has an adaptation function value and speed to adjust its flight direction to ensure that the particle flies toward the food. During the flight, all particles in the group can memorize where they have been, adjust their position, and understand the best position they have experienced [36]. To achieve the optimal solution, each particle must approach the food by constantly learning from the best position it has experienced (pbest) and the best particle position in the population (gbest). The process of learning from pbest is called self-aware part learning, and the process of learning from gbest is called social part learning. Figure 2 shows a schematic of the adjustment of the particle velocity and position in the t and t+1 generations, with the global optimal at X. Here, $v_{1}$ is the speed at which the social part learning causes the particle to fly in the direction of gbest at the iteration moment t, $v_{2}$ represents the speed at which the self-aware part learning causes the particle to fly in the direction of pbest at the iteration moment t, and $v_{3}$ represents the velocity of the particle itself. Under the combined action of $v_{1}$, $v_{2}$, and $v_{3}$, the final particle reaches the new particle position $x_{t+1}$ at $v_{t+1}$. At the next moment, the particle continues to iterate from position $x_{t+1}$, moving closer to the optimal position X with the same synthesis way of speed and position [37].

**Figure 3:**
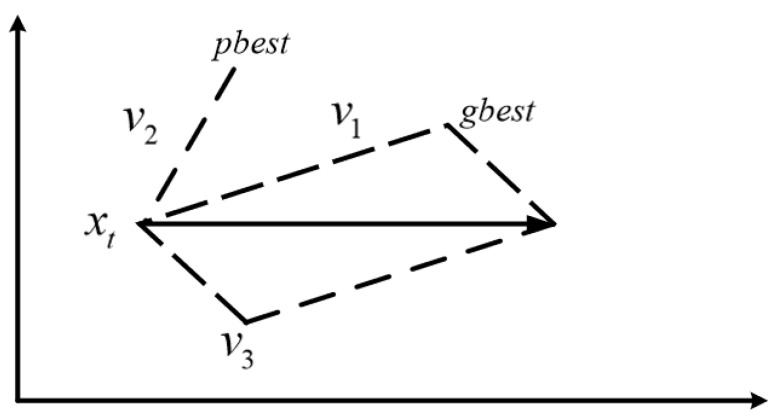
Position and velocity of particles before the update.

**Figure 4:**
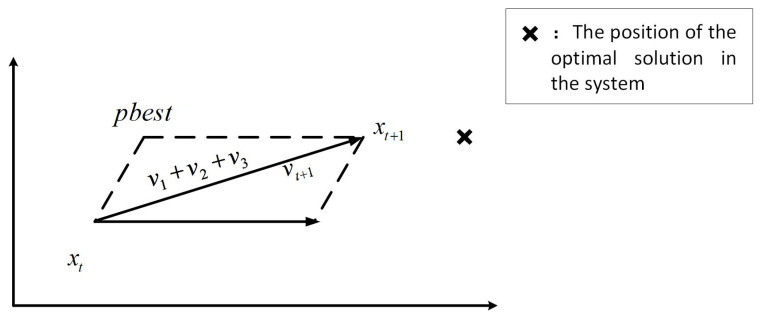
Updated position and velocity of particles.

In a multidimensional spatial coordinate system, the mathematical description of particle swarm operation is as follows: the population size of the particle swarm is N. A single particle i in a population is represented as $x_{i}=(x_{i1},x_{i2},\ldots ,x_{id},\ldots ,x_{iD})$ in d-dimensional space, and its speed is expressed as $v_{i}=(v_{i1},v_{i2},\ldots ,v_{id},\ldots ,v_{iD})$. Then, the flight speed $v_{id}$ of particle i at t+1 moment in the d-dimensional subspace can be expressed as follows:

(5) $$v_{id}(t+1)=v_{id}(t)+c_{1}r_{1}*(p_{id}(t)-x_{id}(t))+c_{2}r_{2}*(p_{gd}(t)-x_{id}(t))$$

(6) $$x_{id}(t+1)=x_{id}(t)+v_{id}\left(t+1\right)$$

(7) $$\left\{\begin{array}{c} v_{id}=v_{\max},if\;v_{id}>v_{\max}\\ v_{id}=-v_{\max},if\;v_{id}<-v_{\max} \end{array}\right.$$

where $c_{1}$ and $c_{2}$ are the learning factors of the particle swarm and are prespecified constants, $r_{1}$ and $r_{2}$ are random numbers generated by a random function at [0, 1], $p_{id}$ represents the optimal position of the particle at its current moment, and $p_{gd}$ stands for the historical best position of the population, that is, the global optimal solution [38]. Since particles moving in the search space may exceed the original maximum velocity after their velocity is updated, Formula (7) is used to limit the velocity of the particle after it is updated. The particle velocity and position before and after updates are shown in Figure 3 and Figure 4, respectively.

The PSO algorithm process is described below.

Step 1: At t=0, initialize the position and velocity of each particle in the population. Set the maximum velocity $v_{\max}$, set the evaluation function of the particle, randomly generate m particles $x_{1},x_{2},\ldots ,x_{m}$ in the defined space to form the initial population X(t), and generate the initial velocity $v_{1},v_{2},\ldots ,v_{m}$ of the particles, thus forming the velocity matrix V(t). The $p_{best}$ of each particle is its initial position, and $g_{best}$ is the best $p_{best}$ of all particles.

Step 2: Calculate the fitness value for each particle.

Step 3: Compare the adaptation value of each particle with the adaptation value of $p_{best}$ and the best $g_{best}$ of the population. Update the individual optimal position and the overall optimal position of each particle.

Step 4: Update the velocity and position of each particle according to Equations (5)–(7).

Step 5: Check whether the termination condition is met. If the set conditions are met, the iteration is terminated; the termination condition is generally to reach the maximum number of iterations. If the termination condition is not met, return to Step 2.

### 3.3. Model for Sensory Evaluation of Blueberries Based on PSO-SVM

**Figure 5:**
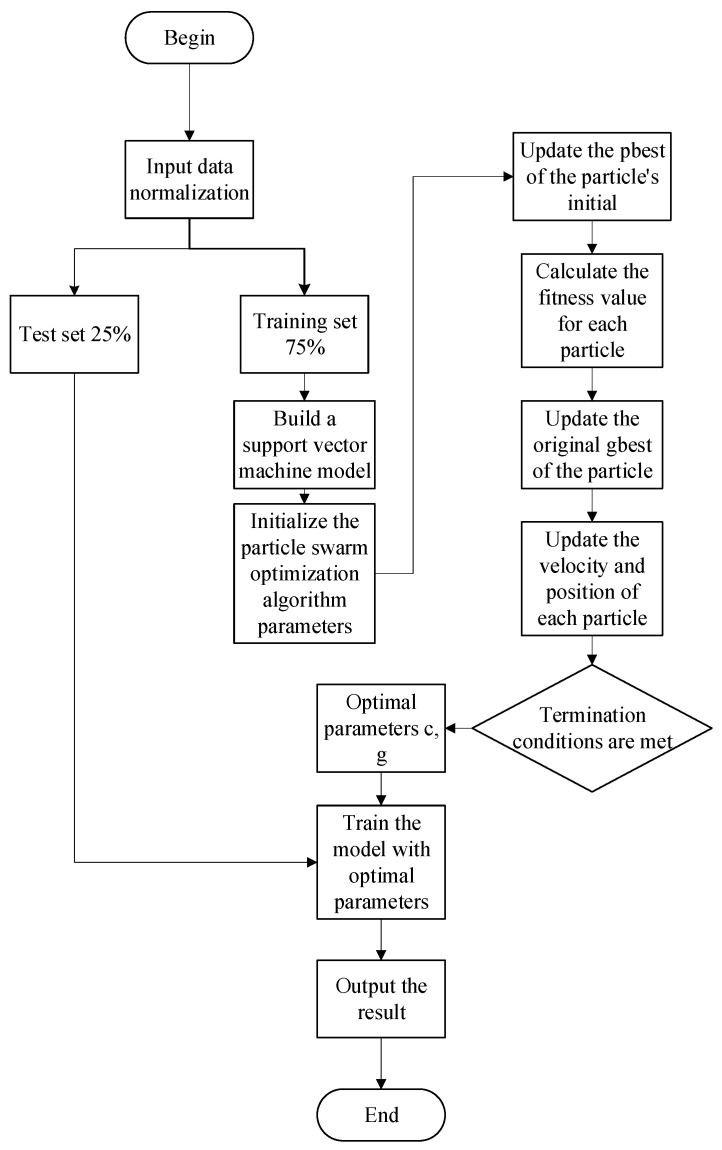
Establishment of a blueberry sensory evaluation prediction model based on particle swarm optimization and a support vector machine.

In this paper, a blueberry sensory evaluation prediction model is proposed, which uses PSO to optimize the parameters of the SVM to improve prediction accuracy. The entire process is compiled and implemented in MATLAB2018b. The implementation of the SVM uses the LIBSVM toolbox, selects the radial basis function as the core function, and then continuously iterates through the PSO algorithm until it reaches the termination state. In this way, we obtain the two key parameters that determine the accuracy of the SVM model: the penalty factor c and the kernel function parameter g. Finally, the test set is used to evaluate the accuracy of the model. The entire model is shown in Figure 5.

## 4. Results

In this paper, we used 75% of the data as the training set and 25% as the test set. An SVM optimized by the particle swarm algorithm (PSO–SVM) was used to construct a predictive model for blueberry sensory evaluation.

The model’s performance was evaluated using three indicators: root mean squared error (RMSE), mean absolute error [39] (MAE), and R^2^.

The root mean square error is the square root of the square sum of the deviation between the predicted value and the true value and the ratio of the number of predictions n. It is used to measure the deviation between the predicted value and the true value; the smaller the root mean square error, the higher the measurement accuracy. The average absolute error is the average of the absolute value of all the individual predicted values and the deviation of the arithmetic mean, and it is used to measure the distance between the predicted value and the true value. It can accurately express the size of the prediction error. R^2^ is a relative metric whose main role is to normalize results and make it easier to identify differences between models; thus, it can be used for comparing models trained on the same data [40].

The formulas for these three indicators are as follows:

(8) $$\mathrm{RMSE}=\sqrt{\frac{{\sum_{i=1}^{n}\left(y_{i}-{\hat{y}}_{i}\right)}^{2}}{n}}$$

(9) $$\mathrm{MAE}=\frac{1}{n}\sum_{i=1}^{n}\left|y_{i}-{\hat{y}}_{i}\right|$$

(10) $$R^{2}=1-\frac{\sum_{i=1}^{n}{\left({\hat{y}}_{i}-{\bar{\hat{y}}}_{i}\right)}^{2}}{\sum_{i=1}^{n}{\left(y_{i}-{\bar{y}}_{i}\right)}^{2}}\in [0,1]$$

where n is the sample number, $\hat{y}$ is the true value of the blueberry sensory evaluation, $\bar{\hat{y}}$ is the average of the true value of blueberry sensory evaluations, y is the predicted result, and $\bar{y}$ is the average of the predicted results. Table 3 describes the process of establishing the PSO-SVM prediction model.

**Figure 6:**
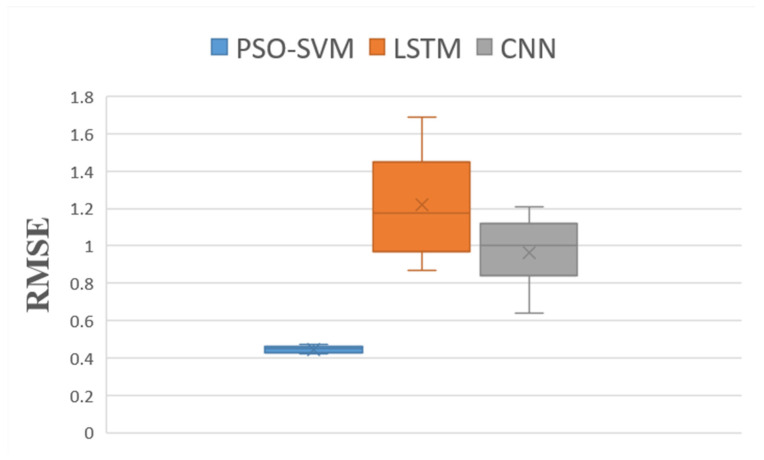
Distribution boxplot of RMSE data for each model.

**Figure 7:**
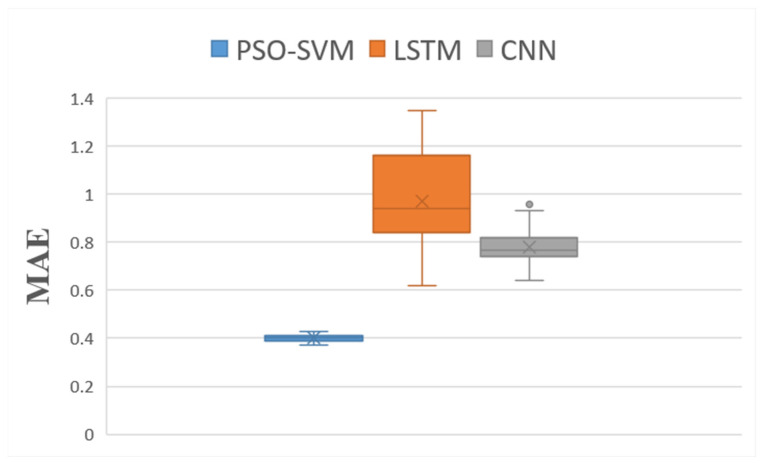
Distribution boxplot of MAE data for each model.

**Figure 8:**
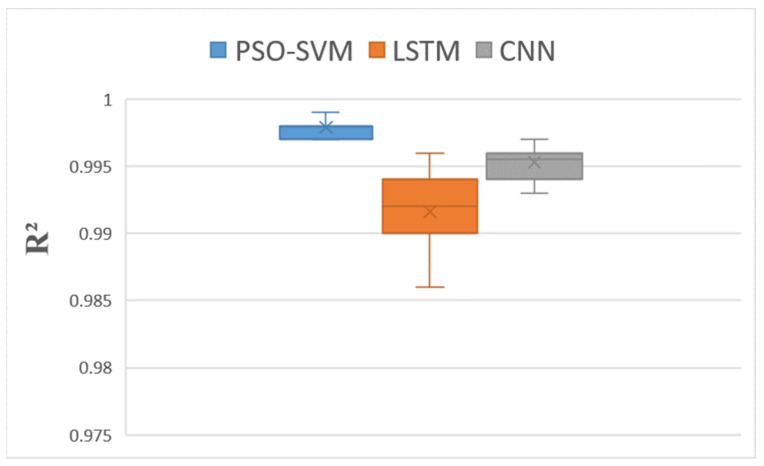
Distribution boxplot of R^2^ data for each model.

A convolutional neural network (CNN) [41] model and long short-term memory [42] (LSTM) model were set up for comparison. The number of iterations was consistent across all the experiments. We used the CNN and LSTM models to compare the accuracy of our model to those of machine learning and deep learning models for small sample data regression problems. To fully verify the validity of the proposed model, each experiment was independently repeated 20 times to ensure the objectivity of the results. The statistical results are plotted as box plots in Figure 6, Figure 7 and Figure 8.

**Figure 9:**
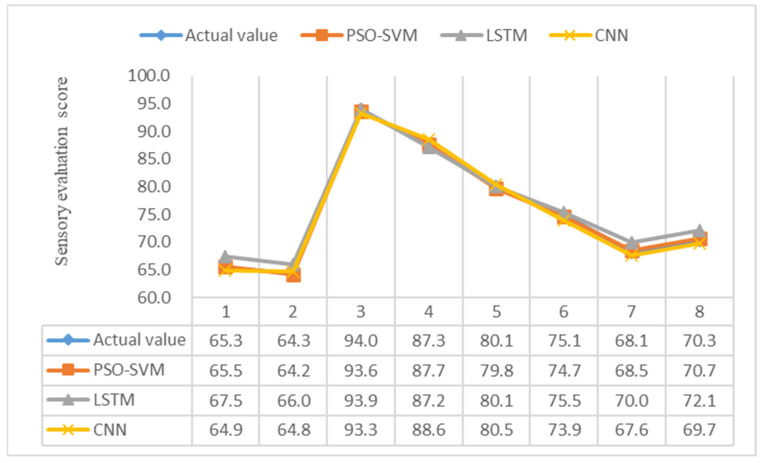
Blueberry sensory evaluation prediction scores for each model.

As can be seen in Figure 6 and Figure 7, the RMSE and MAE boxes of the PSO-SVM model are smaller than those of the CNN and LSTM models. The average RMSE of the PSO-SVM model was 0.45, and the average MAE was 0.40. The mean RMSE for the LSTM model was 1.22, and the mean MAE was 0.97. The CNN model had a mean RMSE of 0.96 and a mean MAE of 0.78. These results prove that the PSO-SVM model is more accurate than the other two models in small-sample regression problems. It can be seen in Figure 8 that the R^2^ box of the PSO-SVM model is smaller than those of the LSTM and CNN models. This proves that the fit of our model is the best. In Figure 9, it can be seen that among the three models for predicting the blueberry sensory evaluation score line, the line predicted using the LSTM model had the largest deviation from the actual value, followed by the CNN model; however, the line predicted using the PSO-SVM model was closest to the actual value. Hence, the PSO-SVM model, as a machine learning model, has higher accuracy and stability than the other two deep neural network models in small-sample regression problems.

The PSO-SVM-based blueberry sensory evaluation prediction model may have a positive impact on the entire blueberry industry chain and related stakeholders in the following ways: (1) Increasing farmer income: By predicting the quality of blueberries in a timely manner, farmers can better manage and sell their products. They can optimize harvest and sale times based on forecasts, maximizing the price of products and market demand and thereby increasing revenue. (2) Reducing food waste: Blueberries are a perishable fruit, and improper storage and sale can lead to significant food waste. Based on this evaluative predictive model, the freshness and shelf life of blueberries can be more accurately judged, helping to reduce food waste and thus contributing to the achievement of the Sustainable Development Goals. (3) Enhancing industry competitiveness: The blueberry industry is becoming increasingly competitive, requiring not only high-quality products, but also efficient and high-tech management capabilities. By adopting the blueberry sensory evaluation prediction model based on PSO-SVM, enterprises can predict market demand and product quality in advance, better meet the needs of consumers, and enhance the competitiveness of the industry. (4) Supporting sustainable agriculture: Blueberries are an important crop, and the environmental and resource impact of their production process cannot be ignored. By applying this sensory evaluation predictive model, blueberry loss and waste can be reduced, thereby reducing the demand for land, water, and energy and contributing to the achievement of agricultural sustainability goals.

## 5. Conclusions

Comparative experiments have shown that in problems involving small samples, the SVM model is more applicable than neural network models, as the number of parameters of the neural network models far exceeds the sample size. Due to the strong global optimization ability and fast convergence speed of the PSO algorithm, the results of our blueberry sensory evaluation prediction model were the best of the three models tested. 

Overall, the sensory evaluation prediction model of blueberries based on PSO-SVM can help improve farmers’ revenue, reduce food waste, promote industry competitiveness, and support sustainable agriculture. This will bring positive economic, social, and environmental benefits to the entire blueberry value chain.

We note here that the PSO algorithm requires adjustment of fewer parameters, is based on a simple principle, and it is easy to implement. In the future research, we can use the model to test the physical and chemical indices of different types of food, as well as conduct a more in-depth analysis of the intelligent search strategy, inertial factor, learning factor, and other important parameters of PSO. Hence, we can continue to utilize the potential of this algorithm and further improve the model structure. Therefore, the SVM model is suitable for predicting blueberry sensory evaluation and can also be extended to other small sample fields.

## Figures and Tables

**Table 1:** Sensory evaluation scoring form.

Name	Date					
Prompt		1. The purpose of this review: to distinguish and compare blueberry fruits under different conditions				
		2. Please evaluate from left to right, and score each sample according to the following five items				
		3. Rest for 15 s before evaluating the next sample				
Evaluation indicator/serial number		Appearance	Hardness	Color	Aroma	Taste

Appearance: rot 0–5; severe water loss in fruit peel 5–10; fruit peel fold 10–15; the fruit is plump and complete 15–20; hardness: soft 0–5; lower hardness 5–10; moderate hardness 10–15; high hardness 15–20; color: not good 0–40; general 40–60; better 60–80; very good 80–100; aroma: light aroma 0–5; aroma generally 5–10; the aroma is more intense 10–15; aroma is rich 15–20; taste: thin 0–40; general 40–60; fuller and rounder 60–80; plump and rounded 80–100.

**Table 2:** Physical and chemical indices and sensory evaluation scores of select samples.

Physical and Chemical Indexes/Sensory Evaluation	Sample				
	1	2	3	4	5
PPO (U/g)	3.02	5.21	6.01	5.86	5.55
APX (U/g)	9.21	10.03	8.12	7.69	5.86
SOD (U/g)	12.31	11.46	10.37	9.28	8.77
POD (U/g)	70.20	74.31	81.48	80.24	75.81
CAT (U/g)	112.69	114.64	115.07	114.21	113.86
Sensory evaluation score	93.97	83.16	75.26	67.21	61.13

**Table 3:** The process of establishing the PSO-SVM predictive model.

Model Building Steps	Detailed Process
1. Data preprocessing	(1) Read the data from an Excel file, save it in the variable data, and then normalize it. (2) Randomly divide the data into training and test sets at a 3:1 ratio to avoid the influence of ordinal data on the model. (3) Extract input and output data from training and test sets
2. PSO optimization process	(1) Set the number of particle swarms to 50 and the number of iterations to 100. Set the ac-celeration constants c1 = 2, c2=2, and inertia weight w = 0.7. Set the maximum velocity $v_{\max}$ according to expert experience, and initialize the particle swarm. (2) Set the evaluation function of the particle, randomly generate m$\;\mathrm{particles}\;x_{1},x_{2},\ldots ,x_{m}$ in the defined space to form the initial population X(t), and generate the initial velocity $v_{1},v_{2},\ldots ,v_{m}$ of the particles, thus forming the velocity matrix V(t). The $p_{best}$ of each particle is its initial position, and $g_{best}$ is the best $p_{best}$ of all the particles. (3) Calculate the fitness value for each particle and compare the adaptation value of each particle with the adaptation value of $p_{best}$ and the best $g_{best}$ of the population. Update the individual optimal position and the overall optimal position of each particle. Update the velocity and position of each particle according to Equations (5)–(7). (4) Check whether the termination condition is met. If the set conditions are met, the iteration is terminated. The termination condition is generally to reach the maximum number of iterations. If the termination condition is not met, return to (3). (5) After the iteration, two values from $g_{best}$ are assigned to the SVM model.
3. SVM predictive model training process	(1) Use the radial basis function to map the training input dataset to an inner product matrix in a high-dimensional space using the formula $K(x_{i},x_{j})=\varphi {\left(x_{i}\right)}^{T}\varphi (x_{j})$. (2) The two values obtained after PSO optimization are assigned to c and g. (3) Train the model using the training set data according to Equations (2)–(4).
4. Calculate model performance evaluation metrics	(1) Enter the input data of the test set in the model to obtain the predicted output data. (2) Calculate RMSE, MAE, and R^2^ according to Equations (8)–(10).

## Data Availability

The authors confirm that the data supporting the findings of this study are available within the article.
